# A Genetic Risk Score Improves the Prediction of Type 2 Diabetes Mellitus in Mexican Youths but Has Lower Predictive Utility Compared With Non-Genetic Factors

**DOI:** 10.3389/fendo.2021.647864

**Published:** 2021-03-12

**Authors:** América Liliana Miranda-Lora, Jenny Vilchis-Gil, Daniel B. Juárez-Comboni, Miguel Cruz, Miguel Klünder-Klünder

**Affiliations:** ^1^ Epidemiological Research Unit in Endocrinology and Nutrition, Hospital Infantil de México Federico Gómez, Mexico City, Mexico; ^2^ Pediatric Medical Residency, Hospital Infantil de México Federico Gómez, Mexico City, Mexico; ^3^ Medical Research Unit in Biochemistry, Hospital de Especialidades Centro Médico Nacional SXXI, Instituto Mexicano del Seguro Social, Mexico City, Mexico; ^4^ Research Subdirectorate, Hospital Infantil de México Federico Gómez, Mexico City, Mexico

**Keywords:** type 2 diabetes, children, youth, genetic risk score, risk factors, obesity, body mass index

## Abstract

**Background:**

Type 2 diabetes (T2D) is a multifactorial disease caused by a complex interplay between environmental risk factors and genetic predisposition. To date, a total of 10 single nucleotide polymorphism (SNPs) have been associated with pediatric-onset T2D in Mexicans, with a small individual effect size. A genetic risk score (GRS) that combines these SNPs could serve as a predictor of the risk for pediatric-onset T2D.

**Objective:**

To assess the clinical utility of a GRS that combines 10 SNPs to improve risk prediction of pediatric-onset T2D in Mexicans.

**Methods:**

This case-control study included 97 individuals with pediatric-onset T2D and 84 controls below 18 years old without T2D. Information regarding family history of T2D, demographics, perinatal risk factors, anthropometric measurements, biochemical variables, lifestyle, and fitness scores were then obtained. Moreover, 10 single nucleotide polymorphisms (SNPs) previously associated with pediatric-onset T2D in Mexicans were genotyped. The GRS was calculated by summing the 10 risk alleles. Pediatric-onset T2D risk variance was assessed using multivariable logistic regression models and the area under the receiver operating characteristic curve (AUC).

**Results:**

The body mass index Z-score (Z-BMI) [odds ratio (OR) = 1.7; p = 0.009] and maternal history of T2D (OR = 7.1; p < 0.001) were found to be independently associated with pediatric-onset T2D. No association with other clinical risk factors was observed. The GRS also showed a significant association with pediatric-onset T2D (OR = 1.3 per risk allele; p = 0.006). The GRS, clinical risk factors, and GRS plus clinical risk factors had an AUC of 0.66 (95% CI 0.56–0.75), 0.72 (95% CI 0.62–0.81), and 0.78 (95% CI 0.70–0.87), respectively (p < 0.01).

**Conclusion:**

The GRS based on 10 SNPs was associated with pediatric-onset T2D in Mexicans and improved its prediction with modest significance. However, clinical factors, such the Z-BMI and family history of T2D, continue to have the highest predictive utility in this population.

## Introduction

The prevalence of type 2 diabetes (T2D) throughout Mexico in 2018 was 10.3%, one of the highest globally and rising (1). Although information regarding the national prevalence of pediatric-onset T2D remains unavailable, an increasing proportion of younger individuals have been affected with more aggressive phenotypes nowadays (2–4).

T2D is a multifactorial disease caused by a complex interplay between genetic and environmental risk factors. Multiple non-genetic risk factors have been associated with T2D, including age, perinatal risk factors, ethnicity, family history, low socioeconomic status, obesity, metabolic syndrome components, and unhealthy lifestyle behaviors (5–7). However, genetics has been hypothesized to play a much greater role in the development of T2D among the younger populations (8).

Although genome-wide association and candidate gene studies have identified hundreds of single nucleotide polymorphism (SNPs) associated with T2D, these variants still only explain less than 20% of the disease’s heritability (9–12). While most of the identified SNPs have been associated with relatively low risk for TD2, combining several markers together through a genetic risk score (GRS) may indicate higher risk (13–22).

Some authors have reported that a high GRS was associated with a younger age at T2D diagnosis (23, 24). However, insufficient information has been available regarding whether genetic information improves prediction models for pediatric-onset T2D. Our estimates have shown that pediatric-onset T2D had a heritability of 50% among Mexicans (25). Endeavoring to elucidate this genetic predisposition, our research team has identified 10 SNPs associated with pediatric-onset T2D among Mexicans with low individual risk [odds ratio (OR) between 1.4 and 2.2] (23, 26, 27). Integrating these genetic factors into a GRS to be used in conjunction with classical risk factors for T2D could be a useful approach toward improving prediction models for the disease (12, 14–16, 28).

Given the importance of identifying risk factors for the early development of T2D on its prevention, genetic information could conceivably help identify individuals at high risk for pediatric-onset T2D. The current study therefore sought to evaluate the predictive utility of incorporating a GRS comprising 10 SNPs into a model with clinical risk factors for pediatric-onset T2D among Mexicans.

## Research Design and Methods

This case–control study was conducted at the Children’s Hospital Federico Gómez, Mexico.

### Participants

Patients aged between 8 and 18 years who lived in Mexico City’s metropolitan area, had the last three family generations born in Mexico, and had genetic mosaic proportions of 65% Native American, 30% European, and 5% African were included (29–31).

Cases included participants under 18 years of age who were diagnosed with T2D for less than 1 month according to the following criteria: (a) previous diagnosis and/or oral glucose tolerance test (OGTT) according to the American Diabetes Association criteria (32); (b) absence of anti-glutamic acid decarboxylase and anti-insulin antibodies; (c) no clinical features of maturity-onset diabetes among younger participants; and (d) C-peptide ≥ 0.45 ng/ml.

Controls were recruited by requesting the participation of friends or neighbors who were of similar age and sex but had no family relationship with the index cases (population controls). Control participants were characterized as non-T2D through the OGTT. We excluded participants with impaired fasting glucose and impaired glucose tolerance.

Enrolled participants provided written assent in addition to written consent from their parents. This study had been approved by the local ethics, biosafety, and research committees.

### Clinical Risk Factors

Information regarding demographics and medical history from family pedigrees over three generations were obtained from questionnaires provided by trained research technicians. All participants were asked to answer questions regarding any case of diabetes in their family, relatives age at diagnosis, and treatment and type of diabetes. T2D diagnosis among the parents were corroborated through an OGTT in those with no previous history of T2D. Information regarding birthweight, gestational age, gestational diabetes exposure, and breastfeeding onset and duration were corroborated with information obtained from in the newborn sheet.

Diet was assessed using an adapted version of the semi-quantitative food frequency intake questionnaire from the previous month. As support material, the interviewer used food replicas to standardize the types and amounts of the main food groups consumed by the participants. The questionnaire contained 119 food items classified into 13 groups. Participants’ food intake per day was estimated, after which the amount food consumed was measured in terms of units (e.g., piece, cup, plate, or spoon) and size (i.e., small, medium, or large). For analysis, consumption frequencies were calculated in grams or milliliters ingested per day for each food item. Energy and macronutrient intake was determined using the Food Processor software (version 10.10, 2012, ESHA Research Inc, Salem, OR), which includes Mexican foods (33). Percentages of adequate energy and macronutrient intake were calculated using recommendations for the Mexican population.

Physical activity was assessed using a physical activity questionnaire, which assessed intensity, frequency, and duration of the physical activities over 1 week. Standard procedures were performed using continuous scales of weekly energy expenditure expressed in metabolic equivalents (MET) × min/day (34).

Fitness was evaluated using a modified Harvard step test, which consisted of stepping onto and off (both feet) a stool 30 cm high, 42 cm wide, and 38 deep for 5 min at 30 cycles per min. Heart rates were recorded at 0, 1, and 2 min after participants finished or prematurely stopped the exercise. The physical fitness score was calculated from the total number seconds the exercise was performed multiplied by 100 and divided by the sum of the 3 heart rate values (35).

### Clinical and Biochemical Variables

Anthropometric data were collected using standard methods. Body height and weight were measured using a column scale with a stadiometer. Weight was determined using a digital scale (Seca^®^ 884, Hamburg, Germany) with an accuracy of 0.1 kg, while height was determined using a stadiometer (Seca^®^ 225, Hamburg, Germany) with a precision of 0.1 cm. Body mass index (BMI) was calculated as weight in kilograms divided by the square of height in meters, after which the BMI Z-score (Z-BMI) was calculated according to age and sex, taking the 2007 WHO data as reference (35). Waist circumference (WC) was measured at the midpoint between the lower costal border and the iliac crest during the end of exhalation using a non-elastic flexible tape to the nearest 0.1 cm (Seca^®^ 200) in a standing position.

Blood samples were drawn after an overnight fast to measure glucose (hexokinase method Dimension RXL.MAX, Siemens), insulin (chemiluminescence IMMULITE 1000, Siemens, Euro, DPC, Llanberis, UK), C-peptide (chemiluminescence IMMULITE 1000, Siemens, Euro, DPC, Llanberis, UK), and hemoglobin A1c (Dimension RXL.MAX Siemens immunoassay). Participants without diabetes underwent an OGTT with 1.75 g/kg of anhydrous glucose (up to 75 g), with glucose and insulin being measured 2 h after glucose administration. To evaluate insulin resistance, the homeostatic model assessment for insulin resistance (HOMA-IR) was calculated using the following formula: HOMA-IR = fasting insulin (μU/mL) × fasting glucose (mg/dl)/405 (36).

### Genotyping

SNPs were selected considering previously published data suggest their association with pediatric-onset T2D in Mexicans (**Table 1**). DNA was extracted from peripheral blood leukocytes using commercial kits following the manufacturer’s instructions (QIAmp96 DNA Blood, Mini/Kit, Qiagen, Germany). Purity and concentration were assessed through spectrophotometry at 260/280 nm (Epoch spectrophotometer, BioTek Instruments, Winooski, VT, USA), and the integrity was checked following 0.8% agarose gel electrophoresis. SNPs were genotyped using the TaqMan^®^ OpenArray^®^ system (Applied Biosystems, Foster City, CA, USA).

**Table 1:** List of the 10 single nucleotide polymorphism considered in the genetic risk score computation.

Reported gene	SNP	Functional class	Alleles	MAF (%) Mexicans	MAF (%) 1000 GP (37)	OR	95% CI	p	Reference
*SLC16A11*	rs13342232	synonymous	A/G	41.6	16.3	1.9	1.2-3.0	0.003	(26)
*ADORA1*	rs903361	intergenic	A/G	27.8	37.7	1.9	1.2-3.0	0.010	(27)
*CADM2*	rs13078807	intron	A/G	9.3	8.7	2.2	1.2-4.0	0.009	(27)
*GNPDA2*	rs10938397	intergenic	A/G	32.5	32.6	2.2	1.4-3.7	9.0E-4	(27)
*VEGFA*	rs6905288	downstream	G/A	35.5	35.5	1.4	1.1-2.1	0.044	(27)
*FTO*	rs9939609	intron	T/A	20.6	34.0	1.8	1.0-2.3	0.039	(27)
*POC5*	rs2112347	upstream	G/T	29.6	48.9	1.7	1.4-2.1	6.9E-4	(23)
*RPS10*	rs206936	intron	A/G	34.6	35.5	1.5	1.2-1.8	0.005	(23)
*GLIS3*	rs7034200	intron	A/C	35.5	44.4	2.1	1.8-2.4	1.9E-6	(23)
*LINGO*	rs10968576	intron	A/G	21.2	20.4	2.0	1.6-2.4	4.3E-4	(23)

SNP, single nucleotide polymorphism; MAF, minor allele frequency; 1000 GP, 1000 genome project; OR, odds ratio; CI, confidence interval.

### Statistical Analysis

Demographic and clinical characteristics of the participants were examined. Continuous variables were expressed as mean ± standard deviation, whereas categorical variables were expressed as numbers and percentages. Bivariate analyses were performed using Student’s t-test, the Mann–Whitney U, or the Chi-square test according to the type and distribution of the variable.

Allelic and genotypic frequencies were determined, after which genotype distribution was confirmed from the Hardy–Weinberg equilibrium (p > 0.05). Associations between individual SNPs and pediatric-onset T2D were analyzed using logistic regression adjusting for age, gender, and Z-BMI with Bonferroni correction for multiple comparison (p < 0.05).

To examine the cumulative effect of the SNPs, an unweighted GR was computed. Each participant was assigned 0, 1, or 2 points according the number of risk alleles for each SNP. The GRS was then constructed by summing the number of risk alleles, assuming an additive genetic model and equal contribution of each SNP to pediatric-onset T2D. Student’s t-tests were used to compare the GR distribution between groups.

Pediatric-onset T2D risk variance was assessed using multivariable logistic regression models and evaluated McFadden’s pseudo R^2^. To develop the best prediction model for pediatric-onset T2D, univariable analysis for each independent variable was performed, subsequently selecting those with a p value < 0.1 as candidate predictors for the multivariable model. A multivariable model was then constructed using the candidate predictors, with <0.05 indicating significance of the collinearity and backward elimination procedure for the selected predictors. Moreover, age, gender, and fitness score (the unique objective lifestyle variable) were forced into the final model despite not being significant candidates for the multivariable model. The strength of the association was measured using odd ratios (OR) and their 95% confidence intervals (CI). Finally, three models were established: Model 1 comprising only clinical risk factors (age, sex, Z-BMI, maternal diabetes, and fitness score); Model 2 comprising only GRS; and Model 3 comprising only clinical risk factors plus GRS.

The fit of the models was also evaluated using the likelihood ratio test, which compares the difference in area under the receiver operating characteristic curve (AUC) to evaluate the models’ ability to predict pediatric-onset T2D. Models for clinical variables with and without GRS were compared using the Chi-squared test. All analyses were performed using STATA SE v11.0 statistical software (STATA Corp, College Station, TX).

## Results

A total of 97 cases with pediatric-onset T2D and 83 controls were included, with **Table 2** showing the main differences in clinical characteristics between cases and controls. Cases reported more obesity indexes and expectedly higher glucose related traits compared to controls. The proportion of children with a family history of T2D history and exposure to gestational diabetes (GD) was significantly higher among those with pediatric-onset T2D than among controls. Moreover, cases who had already received treatment at the time diagnosis exhibited lower self-reported energy and macronutrient consumption and higher physical activity (METS). However, no difference in fitness scores objectively measured with the modified Harvard step test was noted.

**Table 2:** Clinical characteristics of cases and controls.

Trait	Cases (n = 97)		Controls (n = 83)	
**Sociodemographic and anthropometrics**				
Females n(%)	45 (46.4)		38 (45.2)	
Age (years)	12.9 ± 2.6		12.5 ± 2.9	
Weight (kg)	64.2 ± 20.3		57.15 ± 21.4*	
Height (cm)	157.2 ± 13.3		152.4 ± 14.7*	
BMI (kg/m^2^)	25.3 ± 5.3		24.4 ± 6.7	
Z-BMI	1.5 ± 0.7		1.0 ± 1.1*	
WC (cm)	86.5 ± 15.0		80.2 ± 19.4*	
**Biochemical traits**				
Fasting glucose (mg/dl)	149.6 ± 77.2		89.0 ± 8.5*	
2-h glucose (mg/dl)	–		97.4 ± 19.7	
HbA1c (%)	9.6 ± 3.4		5.6 ± 0.5*	
Insulin (mUI/ml)	15.0 ± 19.6		10.3 ± 9.1*	
HOMA-IR	5.2 ± 0.7		2.3 ± 0.3*	
C-peptide (ng/dl)	2.4 ± 2.2		2.8 ± 2.3	
**Family history of T2D**				
Mother	39 (57.1)		8 (10.3)*	
Father	17 (27.4)		4 (6.9)*	
Maternal grandmother	39 (40.6)		17 (21.0)*	
Paternal grandmother	37 (42.5)		15 (20.3)*	
Paternal grandmother	37 (42.5)		15 (20.3)*	
Maternal grandfather	26 (28.0)		18 (23.4)	
Paternal grandfather	19 (22.1)		19 (25.7)	
**Perinatal factors**				
Birthweight (kg)	3.3 ± 0.6		3.1± 0.5	
Gestational age (weeks)	37.9 ± 1.8		37.7 ± 1.8	
Exposure to GD n (%)	13 (14.1)		1 (1.3)*	
Breastfeeding (months)	9.5 ± 9.8		7.4 ± 5.7	
**Lifestyle**				
kcal/day	1783 ± 910		2316 ± 1152*	
% Carbohydrates	54.5 ± 8.1		54.9 ± 8.8	
% Lipids	27.2 ± 8.1		30.5 ± 7.2*	
Physical activity (METS)	4.2 ± 5.2		2.6 ± 4.6*	
Fitness score	72.3 ± 24.7		74.1 ± 23.7	

Data are means (SD) or counts (percentages), as appropriate.

*p < 0.05 according to type and distribution of the variable: Student’s t test, Mann Whitney U or X^2^.

BMI, body mass index; Z-BMI, Z-score BMI; WC, waist circumference; GD, gestational diabetes.

**Figure 1:**
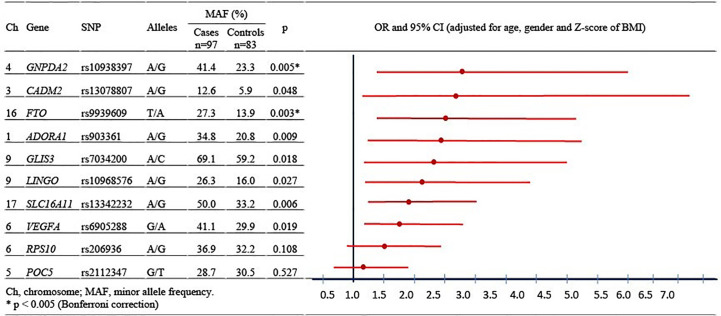
Allele frequency and association between individually single nucleotide polymorphisms and pediatric-onset type 2 diabetes.

All genotyped SNPs were in Hardy-Weinberg equilibrium and had an effect in the expected direction. The additive logistic regression model adjusted for age, gender, and Z-BMI showed that two SNPs were significantly associated with pediatric-onset T2D after Bonferroni correction, while and six SNPs revealed a nominal association with pediatric-onset T2D, yielding ORs ranging from 1.2 to 2.7 (**Figure 1**).

**Figure 2:**
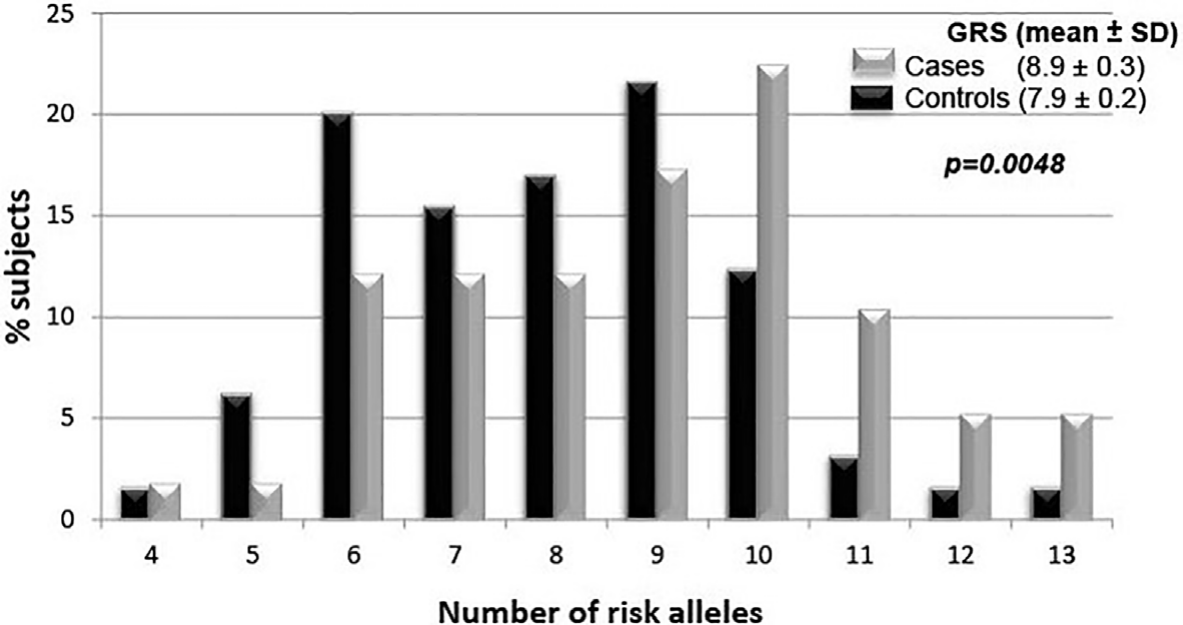
Genetic risk score density of the 10 single nucleotide polymorphisms between cases and controls.

To examine the cumulative effects of the SNPs, the GRS were compared between both groups. Accordingly, pediatric-onset T2D cases had a significantly higher mean GRS value than controls (**Figure 2**).

A multivariable logistic regression model was fitted with all non-genetic factors evaluated. After model reduction, Z-BMI (OR = 1.7; p = 0.009) and maternal history of T2D (OR = 7.1; p < 0.001) were identified as significant and independent clinical predictors of pediatric-onset T2D (Model 1). No association with any other clinical factor was observed. Moreover, Model 2 showed that the GRS was significantly associated with pediatric-onset T2D (OR 1.3 per risk allele; p = 0.006). Finally, Model 3 revealed the same GRS size effect after adjusting for Z-BMI, maternal diabetes, age, gender, and fitness score (**Table 3**).

**Table 3:** Logistic regression for pediatric-onset type 2 diabetes using clinical risk factors and the genetic risk score as predictors.

	OR	95% CI	p
Model 1 (pseudo R^2 ^= 0.15, p < 0.001)*			
BMI (Z-score)	1.7	1.1 ; 2.5	0.009
Maternal Diabetes	7.1	2.8 ; 17.8	<0.001
Age (years)	1.1	0.9 ; 1.2	0.444
Gender (male)	1.1	0.6 ; 2.3	0.72
Fitness score	0.9	0.9 ; 1.0	0.822
**Model 2 (pseudo R^2^ = 0.14, p < 0.001)**			
GRS (per risk allele)	1.3	1.1 ; 1.6	0.006
Model 3 (pseudo R^2^ = 0.21), p < 0.001)*			
GRS (per risk allele)	1.3	1.0 ; 1.6	0.025
BMI (Z-score)	1.5	0.9 ; 2.4	0.146
Maternal Diabetes	8.5	2.8 ; 26.1	<0.001
Age (years)	1.1	0.9 ; 1.3	0.332
Gender (male)	1.2	0.5 ; 2.9	0.75
Fitness score	1.0	1.0 ; 1.0	0.778

*McFadden’s pseudo R^2^, likelihood ratio p < 0.001.

OR, odds ratio; CI, confidence interval.

The McFadden’s test (**Table 3**) showed that the model including only clinical variables and only GRS had a pseudo R^2^ of 0.15 and 0.14, respectively. Notably, the model that included clinical variables plus GRS significantly increased the model fit to 0.21 (p < 0.01).

**Figure 3:**
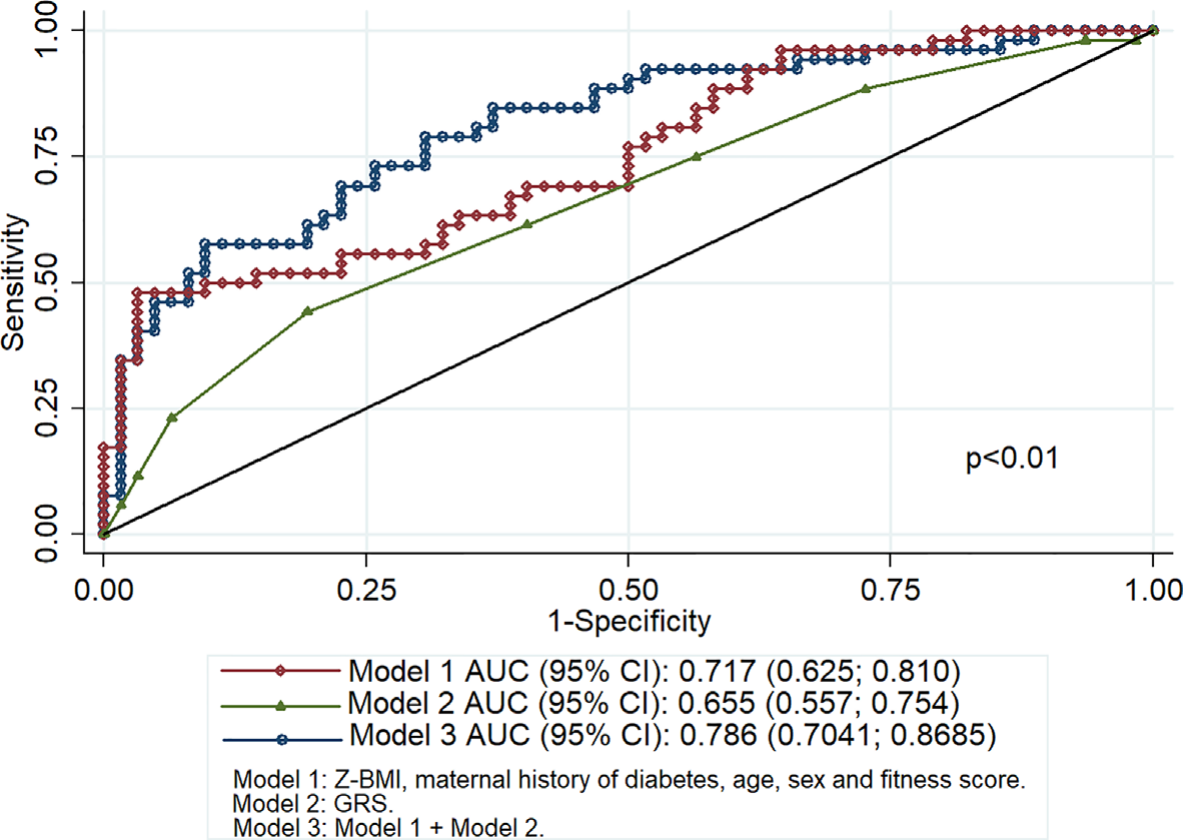
Area under the receiver operating characteristic curve for pediatric-onset type 2 diabetes.

**Figure 3** shows the AUC for pediatric-onset T2D according to the three models. Model 3 had a better performance than Model 1, which in turn had a better performance than Model 2 (p = 0.01). The addition of the GRS into the clinical factors increased the AUC by 7 percentage points.

## Discussion

Developing prediction models for the identification of individuals at risk for early onset T2D is important in order to establish measures for preventing or delaying disease onset. Recently, several studies have shown that certain SNPs were associated with pediatric-onset T2D (23, 26, 27, 36, 38–40). Given that such SNPs individually have low predictive ability for the risk of T2D, the GRS provides an opportunity to evaluate the cumulative effects of genetic factors. Although a previous study had shown that the GRS was associated with younger age at T2D diagnosis (41), to the best of our knowledge, this has been the first study to evaluate the utility of the GRS for predicting pediatric-onset T2D among the Mexican population. Our results are important to determine the utility of genetic factors for identifying susceptible populations in low- to middle-income countries.

The current study found that more than 95% of pediatric-onset T2D cases had six or more risk alleles and a higher mean GRS compared to controls. Although the risk per individual allele was low (OR 1.3, CI95%: 1.1;1.6), combining the markers could indicate great risk. These results are in agreement with the findings of other cross-sectional and follow-up studies wherein different GRSs were associated with T2D-related traits in adults of different ethnic groups (OR 1.06 to 2.2 per risk allele). However, most of the aforementioned studies included tens to hundreds of susceptibility loci (14–16, 27, 28, 42–53). Taken together, the presented information supports the utility of the GRS in prediction models for T2D independent of environmental risk factors.

The present study determined the utility of GRS in improving risk prediction models. Accordingly, out results showed that the model including only clinical variables (Z-BMI, maternal diabetes, age, gender, and fitness score) had pseudo R^2^ value of 15%, whereas incorporating the GRS increased this to 21%. Abdullah et al. previously reported that the inclusion of a GRS promoted an approximately 1%–2% increase in pseudo R^2^ (54).

Our findings showed that while the GRS had a lower predictive ability for pediatric-onset T2D compared to clinical factors, combining both factors prompted a modest yet significant increase. The GRS utilized herein had a better AUC (0.66) compared to that employed in previous reports wherein a GRS with <10 SNPs had AUCs ranging from 0.56 to 0.59, a GRS with 10 to 20 SNPs had AUCs of 0.55 to 0.68, and a GRS with >30 SNPs had AUCs from 0.58 to 0.64 (55). On the other hand, conventional risk models that included variables, such as age, sex, BMI, physical activity level, family history of diabetes, ethnicity, smoking status, alcohol consumption, waist circumference, waist-to-hip ratio, and blood pressure, have reported AUCs ranging from 0.63 to 0.96 (21). The aforementioned findings are similar to those presented herein, where an AUC of 0.72 was achieved for the model including Z-BMI, maternal history of T2D, age, sex and fitness score.

Previous studies have reported that the inclusion of genetic markers resulted in a slight improvement, with differences in AUCs ranging from 0 to 0.12 and net reclassification of T2D risk prediction models from −2.2% to 10.2% (21). However, the current study obtained a 7 percentage point increase in the AUC after combining the GRS and clinical factors, a finding consistent with that reported in previous studies wherein an approximately 1 to 6 percentage point increase in AUCs for adult-onset T2D and gestational diabetes was noted after adding the GRS (27, 45, 54, 56–60). Larger studies have reported that a GRS promoted a considerable improvement in the discrimination of incident T2D, with Talmud et al. (61) showing an 8.1% net reclassification improvement with GRS and Läll y colleagues (62) reporting a 32.4% improvement. Although increasing the number of SNPs included in the GRS increase could improve its accuracy, this would come at increased cost and model complexity.

Specific information for pediatric populations has remained scarce, with previous reports failing to investigate the utility of incorporating the GRS into prediction models. Vassy et al. evaluated models for incident T2D using risk factors assessed in adolescence (demographics, family history, physical examination, and biomarkers) in conjunction with 38 SNPs. Although their findings showed a hazard ratio of 1.06–1.09 per risk allele, the addition of the GRS did not improve the discriminative ability of the model (63). Pitkänen et al., who examined whether the addition of a weighted GRS based on 73 genetic variants to childhood risk factors improved the identification of T2D risk during adulthood, reported a lower and non-significant net reclassification improvement for T2D (2.1%, p = 0.158) (64).

Genetic risk factors, which can be measured objectively, remain unchanged throughout the course of life. However, genotyping carries far greater costs compared to the measurement of conventional risk factors, which, in most cases, requires only a medical history and physical examination. Despite the improvement in pediatric-onset risk classification after including a GRS, the modest effects compared to non-genetic risk factors (Z-BMI and maternal diabetes history) need to be considered, with insufficient evidence to recommend incorporating a GRS into clinical practice. In other words, despite the substantial relevance of a GRS for research studies, it provides limited clinical value to routine medical practice given its modest benefits in improving the prediction of T2D over traditional clinical risk factors. Other authors have reported similar results wherein genetic information provided no incremental value compared to standard non-invasive and metabolic markers, such as age, positive family history, and obesity (65).

A family history of diabetes and, in specific cases, a maternal history of diabetes reflects not only genetic predisposition but also shared environmental and lifestyle factors and even inclusive fetal programming (25, 66). In fact, Do and colleagues showed that a complete family history provides better prediction than 21 SNPs (67). Studies have indicated that a family history of T2D remains a strong, independent, and easily assessed risk factor for T2D (68).

Most SNPs included herein have also been associated with obesity. However, as reported by other authors, we observed that a genetic predisposition to obesity leads to increased risk for T2D independent of Z-BMI (69). Other authors that have evaluated the GRS in conjunction with non-genetic risk factors (obesity, unhealthy life style, family history of T2D among first-degree relatives, and socioeconomic status) have confirmed that latter lead to increased risk of T2D (52, 70–72). These observations highlight the need for prioritizing the prevention of environmental exposure over unmodifiable genetic factors. The main point that needs to be highlighted is that lifestyle interventions have been proven effective in preventing or delaying the T2D onset and could attenuate the effect of the genetic variants (21, 73–81). In addition, individuals who gain weight may be more susceptible to the cumulative impact of T2D variants (14).

The current study evaluated environmental risk factors in conjunction with a GRS, including SNPs previously reported to be associated with pediatric-onset T2D in Mexicans (23, 26, 27). However, the combined model (GRS and clinical factors) only explained 21% of the variance between cases and control. One of the limitations of this study is that our results may not be generalizable to populations with different ethnic backgrounds. Other limitations include the cross-sectional design, which limited our ability to determine the effects of the risk factors on the progression of T2D over time, the small number of individuals included due to the low prevalence of the disease among pediatric patients, the subjective measurements of dietary patterns.

Moreover, we cannot rule out other nonadditive models as an alternative to the unweighted GRS and whether the incorporation of a great number of variants among the hundreds of SNPs associated with T2D might enhance prediction. Furthermore, we could not confirm the association between dietary habits and the risk of pediatric-onset T2D. We believe that the case–control design prevented us from identifying dietary habits that increased risk given that nutritional management for the cases was established at the time of diagnosis. Nonetheless, follow-up studies could perhaps observe the effects of dietary habits on T2D risk as previously reported (82, 83). Despite the lack of association between dietary habits and T2D herein, the involvement of diet is obvious given that it is the main determinant for the Z-BMI. Taken together, larger studies employing a prospective design and including other SNPs and more objective measures of non-genetic factors are certainly needed.

In conclusion, the current study showed that a GRS based on 10 SNPs improved pediatric-onset T2D risk classification in Mexicans after accounting clinical risk factors. The above, is indicative of the clinical potential of adding genetic information in every day clinical practice. However, clinical risk factors, such as maternal history of diabetes and Z-BMI, which can be easily measured in clinical practice, remain the principal risk factors.

## Data Availability Statement

The raw data supporting the conclusions of this article will be made available by the authors, without undue reservation.

## Ethics Statement

The studies involving human participants were reviewed and approved by Comité de Ética en Investigación del Hospital Infantil de México Federico Gómez. Written informed consent to participate in this study was provided by the participants’ legal guardian/next of kin.

## Author Contributions

AM-L and MK-K participated in the conception and design of the study. AM-L, DJ-C, MC, and JV-G contributed to the acquisition of the data. AM-L, MK-K, and JV-G contributed to the analysis and interpretation of the data. AM-L, MK-K, and JV-G wrote the manuscript. All authors contributed to the article and approved the submitted version.

## Funding

This work was supported by the Federal Funds HIM 2014/041.

## Conflict of Interest

The authors declare that the research was conducted in the absence of any commercial or financial relationships that could be construed as a potential conflict of interest.

## References

[1] Shamah-Levy T Vielma-Orozco E Heredia-Hernández O Romero-Martínez M Mojica-Cuevas J Cuevas-Nasu L. Encuesta Nacional de Salud y nutrición 2018-19: Resultados nacionales. Cuernavaca, México: Instituto Nacional de Salud Pública (2020).

[2] Jiménez-Corona A Rojas R Gómez-Pérez FJ Aguilar-Salinas CA. Early-onset type 2 diabetes in a Mexican survey: Results from the National Health and Nutrition survey 2006. Salud Publ Mex (2010) 52(Suppl 1):S27–35. 10.1590/s0036-36342010000700006 20585726

[3] Guerrero-Romero F Violante R Rodríguez-Morán M. Distribution of fasting plasma glucose and prevalence of impaired fasting glucose, impaired glucose tolerance and type 2 diabetes in the Mexican paediatric population. Paediatr Perinat Epidemiol (2009) 23:363–9. 10.1111/j.1365-3016.2009.01035.x 19523083

[4] Lerman-Garber I Aguilar-Salinas C Tusié-Luna T Velásquez D Lobato-Valverde M Osornio-Flores M. [Early-onset type 2 diabetes mellitus. The experience from a third level medical institution]. Gac Med Mex (2010) 146:179–84. 20957814

[5] Kyrou I Tsigos C Mavrogianni C Cardon G Van Stappen V Latomme J. Sociodemographic and lifestyle-related risk factors for identifying vulnerable groups for type 2 diabetes: A narrative review with emphasis on data from Europe. BMC Endocr Disord (2020) 20(Suppl):134. 10.1186/s12902-019-0463-3 PMC7066728 32164656

[6] Juonala M Jääskeläinen P Sabin MA Viikari JS Kähönen M Lehtimäki T. Higher maternal body mass index is associated with an increased risk for later type 2 diabetes in offspring. J Pediatr (2013) 162:918–23.e1. 10.1016/j.jpeds.2012.10.062 23260097

[7] Morrison JA Glueck CJ Wang P. Childhood risk factors predict cardiovascular disease, impaired fasting glucose plus type 2 diabetes mellitus, and high blood pressure 26 years later at a mean age of 38 years: The Princeton-lipid research clinics follow-up study. Metabolism (2012) 61:531–41. 10.1016/j.metabol.2011.08.010 PMC3324938 22001337

[8] Langenberg C Sharp SJ Franks PW Scott RA Deloukas P Forouhi NG. Gene-lifestyle interaction and type 2 diabetes: The EPIC interact case-cohort study. PLoS Med (2014) 11:e1001647. 10.1371/journal.pmed.1001647 24845081 PMC4028183

[9] Scott RA Scott LJ Mägi R Marullo L Gaulton KJ Kaakinen M. An expanded genome-wide association study of Type 2 diabetes in Europeans. Diabetes (2017) 66:2888–902. 10.2337/db16-1253 PMC5652602 28566273

[10] Mahajan A Go MJ Zhang W Below JE Gaulton KJ Ferreira T. Genome-wide trans-ancestry meta-analysis provides insight into the genetic architecture of type 2 diabetes susceptibility. Nat Genet (2014) 46:234–44. 10.1038/ng.2897 PMC3969612 24509480

[11] Gaulton KJ Ferreira T Lee Y Raimondo A Mägi R Reschen ME. Genetic fine mapping and genomic annotation defines causal mechanisms at type 2 diabetes susceptibility loci. Nat Genet (2015) 47:1415–25. 10.1038/ng.3437 PMC4666734 26551672

[12] Wang X Strizich G Hu Y Wang T Kaplan RC Qi Q. Genetic markers of type 2 diabetes: Progress in genome-wide association studies and clinical application for risk prediction. J Diabetes (2016) 8:24–35. 10.1111/1753-0407.12323 26119161

[13] Doria A Patti ME Kahn CR. The emerging genetic architecture of type 2 diabetes. Cell Metab (2008) 8:186–200. 10.1016/j.cmet.2008.08.006 18762020 PMC4267677

[14] Andersson EA Allin KH Sandholt CH Borglykke A Lau CJ Ribel-Madsen R. Genetic risk score of 46 type 2 diabetes risk variants associates with changes in plasma glucose and estimates of pancreatic beta-cell function over 5 years of follow-up. Diabetes (2013) 62:3610–7. 10.2337/db13-0362 PMC3781471 23835328

[15] Saxena R Elbers CC Guo Y Peter I Gaunt TR Mega JL. Large-scale gene-centric meta-analysis across 39 studies identifies type 2 diabetes loci. Am J Hum Genet (2012) 90:410–25. 10.1016/j.ajhg.2011.12.022 PMC3309185 22325160

[16] Lyssenko V Laakso M. Genetic screening for the risk of type 2 diabetes: Worthless or valuable? Diabetes Care (2013) 36(Suppl 2[Suppl:S120–6]):S120–6. 10.2337/dcS13-2009 PMC3920800 23882036

[17] Layton J Li X Shen C de Groot M Lange L Correa A. Type 2 diabetes genetic risk scores are associated with increased Type 2 diabetes risk among African Americans by cardiometabolic status. Clin Med Insights Endocrinol Diabetes (2018) 11(11):1–9. 10.1177/1179551417748942. 1179551417748942. PMC5757425 29326538

[18] Stančáková A Kuulasmaa T Kuusisto J Mohlke KL Collins FS Boehnke M. Genetic risk scores in the prediction of plasma glucose, impaired insulin secretion, insulin resistance and incident type 2 diabetes in the METSIM study. Diabetologia (2017) 60:1722–30. 10.1007/s00125-017-4313-4 28573393

[19] Rao P Zhou Y Ge SQ Wang AX Yu XW Alzain MA. Validation of Type 2 diabetes risk variants identified by Genome-Wide Association Studies in northern Han Chinese. Int J Environ Res Public Health (2016) 13:1–10. 10.3390/ijerph13090863 PMC5036696 27589775

[20] Pigeyre M Sjaarda J Chong M Hess S Bosch J Yusuf S. ACE and Type 2 diabetes risk: A Mendelian randomization study. Diabetes Care (2020) 43:835–42. 10.2337/dc19-1973 32019855

[21] Echouffo-Tcheugui JB Dieffenbach SD Kengne AP. Added value of novel circulating and genetic biomarkers in type 2 diabetes prediction: A systematic review. Diabetes Res Clin Pract (2013) 101:255–69. 10.1016/j.diabres.2013.03.023 23647943

[22] Fontaine-Bisson B Renström F Rolandsson O The MAGIC Investigators Payne F Hallmans G. Evaluating the discriminative power of multi-trait genetic risk scores for type 2 diabetes in a northern Swedish population. Diabetologia (2010) 53:2155–62. 10.1007/s00125-010-1792-y PMC2931645 20571754

[23] Miranda-Lora AL Molina-Díaz M Cruz M Sánchez-Urbina R Martínez-Rodríguez NL López-Martínez B. Genetic polymorphisms associated with pediatric-onset type 2 diabetes: A family-based transmission disequilibrium test and case-control study. Pediatr Diabetes (2019) 20:239–45. 10.1111/pedi.12818 30652413

[24] de Miguel-Yanes JM Shrader P Pencina MJ Fox CS Manning AK Grant RW. Genetic risk reclassification for type 2 diabetes by age below or above 50 years using 40 type 2 diabetes risk single nucleotide polymorphisms. Diabetes Care (2011) 34:121–5. 10.2337/dc10-1265 PMC3005447 20889853

[25] Miranda-Lora AL Vilchis-Gil J Molina-Díaz M Flores-Huerta S Klünder-Klünder M. Heritability, parental transmission and environment correlation of pediatric-onset type 2 diabetes mellitus and metabolic syndrome-related traits. Diabetes Res Clin Pract (2017) 126:151–9. 10.1016/j.diabres.2017.02.005 28242438

[26] Miranda-Lora AL Cruz M Molina-Díaz M Gutiérrez J Flores-Huerta S Klünder-Klünder M. Associations of common variants in the SLC16A11, TCF7L2, and ABCA1 genes with pediatric-onset type 2 diabetes and related glycemic traits in families: A case-control and case-parent trio study. Pediatr Diabetes (2017) 18:824–31. 10.1111/pedi.12497 28101933

[27] Miranda-Lora AL Cruz M Aguirre-Hernández J Molina-Díaz M Gutiérrez J Flores-Huerta S. Exploring single nucleotide polymorphisms previously related to obesity and metabolic traits in pediatric-onset type 2 diabetes. Acta Diabetol (2017) 54:653–62. 10.1007/s00592-017-0987-9 28401323

[28] Goodarzi MO Palmer ND Cui J Guo X Chen YI Taylor KD. Classification of Type 2 diabetes genetic variants and a novel genetic risk score association with insulin clearance. J Clin Endocrinol Metab (2020) 105:1251–60. 10.1210/clinem/dgz198 PMC7059988 31714576

[29] Martinez-Marignac VL Valladares A Cameron E Chan A Perera A Globus-Goldberg R. Admixture in Mexico City: Implications for admixture mapping of type 2 diabetes genetic risk factors. Hum Genet (2007) 120:807–19. 10.1007/s00439-006-0273-3 17066296

[30] Parra EJ Below JE Krithika S Valladares A Barta JL Cox NJ. Genome-wide association study of type 2 diabetes in a sample from Mexico City and a meta-analysis of a Mexican-American sample from Starr County, Texas. Diabetologia (2011) 54:2038–46. 10.1007/s00125-011-2172-y PMC3818640 21573907

[31] Galanter JM Fernandez-Lopez JC Gignoux CR Barnholtz-Sloan J Fernandez-Rozadilla C Via M. Development of a panel of genome-wide ancestry informative markers to study admixture throughout the Americas. PLoS Genet (2012) 8:e1002554. 10.1371/journal.pgen.1002554 22412386 PMC3297575

[32] American Diabetes Association. 2 Classification and diagnosis of diabetes: Standards of medical care in Diabetes-2020. Diabetes Care (2020) 43(Suppl 1):S14–31-s31. 10.2337/dc20-S002 31862745

[33] Voight BF Scott LJ Steinthorsdottir V Morris AP Dina C Welch RP. Twelve type 2 diabetes susceptibility loci identified through large-scale association analysis. Nat Genet (2010) 42:579–89. 10.1038/ng.609 PMC3080658 20581827

[34] Ainsworth BE Haskell WL Whitt MC Irwin ML Swartz AM Strath SJ. Compendium of physical activities: An update of activity codes and MET intensities. Med Sci Sports Exerc (2000) 32(9):S498–504. 10.1097/00005768-200009001-00009 10993420

[35] World Health Organization. Growth reference data for school-aged children and adolescents of 5-19 years; 2007. http://www.who.int/growthref/en/. 10.2471/BLT.07.043497 PMC2636412 18026621

[36] Dabelea D Dolan LM D’Agostino R Jr. Hernandez AM McAteer JB Hamman RF. Association testing of TCF7L2 polymorphisms with type 2 diabetes in multi-ethnic youth. Diabetologia (2011) 54:535–9. 10.1007/s00125-010-1982-7 PMC3766323 21109996

[37] The 1000 Genomes Project Consortium. A global reference for human genetic variation. Nature (2015) 526:68–74. 10.1038/nature15393 26432245 PMC4750478

[38] Giannini C Dalla Man C Groop L Cobelli C Zhao H Shaw MM. Co-occurrence of risk alleles in or near genes modulating insulin secretion predisposes obese youth to prediabetes. Diabetes Care (2014) 37:475–82. 10.2337/dc13-1458 PMC3898754 24062323

[39] Dubinina IA Chistiakov DA Eremina IA Brovkin AN Zilberman LI Nikitin AG. Studying progression from glucose intolerance to type 2 diabetes in obese children. Diabetes Metab Syndr (2014) 8:133–7. 10.1016/j.dsx.2014.07.002 25127329

[40] Jiang YD Chuang LM Pei D Lee YJ Wei JN Sung FC. Genetic variations in the Kir6.2 subunit (KCNJ11) of pancreatic ATP-sensitive potassium channel gene are associated with insulin response to glucose loading and early onset of Type 2 diabetes in childhood and adolescence in Taiwan. Int J Endocrinol (2014) 2014:983016. 10.1155/2014/983016 25309595 PMC4189766

[41] Zhou K Donnelly LA Morris AD Franks PW Jennison C Palmer CN. Clinical and genetic determinants of progression of type 2 diabetes: A DIRECT study. Diabetes Care (2014) 37:718–24. 10.2337/dc13-1995 PMC4038744 24186880

[42] Shin JH Lee KM Shin J Kang KD Nho CW Cho YS. Genetic risk score combining six genetic variants associated with the cellular NRF2 expression levels correlates with Type 2 diabetes in the human population. Genes Genomics (2019) 41:537–45. 10.1007/s13258-019-00791-0 30767168

[43] Kim M Kim M Huang L Jee SH Lee JH. Genetic risk score of common genetic variants for impaired fasting glucose and newly diagnosed type 2 diabetes influences oxidative stress. Sci Rep (2018) 8:7828. 10.1038/s41598-018-26106-z 29777116 PMC5959868

[44] Qi Q Stilp AM Sofer T Moon JY Hidalgo B Szpiro AA. Genetics of Type 2 diabetes in U.S. Hispanic/latino individuals: Results from the Hispanic Community Health Study/Study of Latinos (HCHS/SOL). Diabetes (2017) 66:1419–25. 10.2337/db16-1150 PMC5399610 28254843

[45] Chikowore T van Zyl T Feskens EJ Conradie KR. Predictive utility of a genetic risk score of common variants associated with type 2 diabetes in a black South African population. Diabetes Res Clin Pract (2016) 122:1–8. 10.1016/j.diabres.2016.09.019 27744072

[46] Yaghootkar H Scott RA White CC Zhang W Speliotes E Munroe PB. Genetic evidence for a normal-weight “metabolically obese” phenotype linking insulin resistance, hypertension, coronary artery disease, and type 2 diabetes. Diabetes (2014) 63:4369–77. 10.2337/db14-0318 PMC4392920 25048195

[47] Hassanali N De Silva NM Robertson N Rayner NW Barrett A Bennett AJ. Evaluation of common type 2 diabetes risk variants in a South Asian population of Sri Lankan descent. PLoS One (2014) 9:e98608. 10.1371/journal.pone.0098608 24926958 PMC4057178

[48] Imamura M Shigemizu D Tsunoda T Iwata M Maegawa H Watada H. Assessing the clinical utility of a genetic risk score constructed using 49 susceptibility alleles for type 2 diabetes in a Japanese population. J Clin Endocrinol Metab (2013) 98:E1667–73. 10.1210/jc.2013-1642 23956346

[49] Villegas R Delahanty R Gao YT Long J Williams SM Xiang YB. Joint effect of genetic and lifestyle risk factors on type 2 diabetes risk among Chinese men and women. PLoS One (2012) 7:e49464. 10.1371/journal.pone.0049464 23185337 PMC3504028

[50] Janipalli CS Kumar MV Vinay DG Sandeep MN Bhaskar S Kulkarni SR. Analysis of 32 common susceptibility genetic variants and their combined effect in predicting risk of Type 2 diabetes and related traits in Indians. Diabetes Med J Br Diabetes Assoc (2012) 29:121–7. 10.1111/j.1464-5491.2011.03438.x 21913964

[51] Qi Q Li H Wu Y Liu C Wu H Yu Z. Combined effects of 17 common genetic variants on type 2 diabetes risk in a Han Chinese population. Diabetologia (2010) 53:2163–6. 10.1007/s00125-010-1826-5 20556352

[52] Cornelis MC Qi L Zhang C Kraft P Manson J Cai T. Joint effects of common genetic variants on the risk for type 2 diabetes in U.S. men and women of European ancestry. Ann Intern Med (2009) 150:541–50. 10.7326/0003-4819-150-8-200904210-00008 PMC3825275 19380854

[53] Inaishi J Hirakawa Y Horikoshi M Akiyama M Higashioka M Yoshinari M. Association between genetic risk and development of Type 2 diabetes in a General Japanese population: The Hisayama study. J Clin Endocrinol Metab (2019) 104:3213–22. 10.1210/jc.2018-01782 30830152

[54] Abdullah N Abdul Murad NA Mohd Haniff EA Syafruddin SE Attia J Oldmeadow C. Predicting type 2 diabetes using genetic and environmental risk factors in a multi-ethnic Malaysian cohort. Public Health (2017) 149:31–8. 10.1016/j.puhe.2017.04.003 28528225

[55] Look AHEAD Research Group. Prospective association of a genetic risk score and lifestyle intervention with cardiovascular morbidity and mortality among individuals with type 2 diabetes: The Look AHEAD randomised controlled trial. Diabetologia (2015) 58:1803–13. 10.1007/s00125-015-3610-z PMC4507276 25972230

[56] Kim SH Lee ES Yoo J Kim Y. Predicting risk of type 2 diabetes mellitus in Korean adults aged 40-69 by integrating clinical and genetic factors. Prim Care Diabetes (2019) 13:3–10. 10.1016/j.pcd.2018.07.004 30477970

[57] Kawai VK Levinson RT Adefurin A Kurnik D Collier SP Conway D. A genetic risk score that includes common type 2 diabetes risk variants is associated with gestational diabetes. Clin Endocrinol (2017) 87:149–55. 10.1111/cen.13356 PMC5533106 28429832

[58] Lyssenko V Jonsson A Almgren P Pulizzi N Isomaa B Tuomi T. Clinical risk factors, DNA variants, and the development of type 2 diabetes. N Engl J Med (2008) 359:2220–32. 10.1056/NEJMoa0801869 19020324

[59] Tam CH Ho JS Wang Y Lam VK Lee HM Jiang G. Use of net reclassification improvement (NRI) method confirms the utility of combined genetic risk score to predict type 2 diabetes. PLoS One (2013) 8:e83093. 10.1371/journal.pone.0083093 24376643 PMC3869744

[60] Kwak SH Choi SH Kim K Jung HS Cho YM Lim S. Prediction of type 2 diabetes in women with a history of gestational diabetes using a genetic risk score. Diabetologia (2013) 56:2556–63. 10.1007/s00125-013-3059-x 24057154

[61] Talmud PJ Cooper JA Morris RW Dudbridge F Shah T Engmann J. Sixty-five common genetic variants and prediction of type 2 diabetes. Diabetes (2015) 64:1830–40. 10.2337/db14-1504 PMC4407866 25475436

[62] Läll K Mägi R Morris A Metspalu A Fischer K. Personalized risk prediction for type 2 diabetes: The potential of genetic risk scores. Genet Med (2017) 19:322–9. 10.1038/gim.2016.103 PMC5506454 27513194

[63] Vassy JL Shrader P Jonsson A Fox CS Lyssenko V Isomaa B. Association between parental history of diabetes and type 2 diabetes genetic risk scores in the PPP-Botnia and Framingham Offspring Studies. Diabetes Res Clin Pract (2011) 93:e76–9. 10.1016/j.diabres.2011.04.013 PMC3156338 21570145

[64] Pitkänen N Juonala M Rönnemaa T Sabin MA Hutri-Kähönen N Kähönen M. Role of conventional childhood risk factors versus genetic risk in the development of Type 2 diabetes and impaired fasting glucose in adulthood: The cardiovascular risk in Young Finns study. Diabetes Care (2016) 39:1393–9. 10.2337/dc16-0167 27298332

[65] Mühlenbruch K Jeppesen C Joost HG Boeing H Schulze MB. The value of genetic information for diabetes risk prediction - Differences according to sex, age, family history and obesity. PLoS One (2013) 8:e64307. 10.1371/journal.pone.0064307 23700469 PMC3658960

[66] Monteiro LJ Norman JE Rice GE Illanes SE. Fetal programming and gestational diabetes mellitus. Placenta (2016) 48(Suppl 1):S54–60-s60. 10.1016/j.placenta.2015.11.015 26724985

[67] Do CB Hinds DA Francke U Eriksson N. Comparison of family history and SNPs for predicting risk of complex disease. PLoS Genet (2012) 8:e1002973. 10.1371/journal.pgen.1002973 23071447 PMC3469463

[68] InterAct Consortium Scott RA Langenberg C Sharp SJ Franks PW Rolandsson O. The link between family history and risk of type 2 diabetes is not explained by anthropometric, lifestyle or genetic risk factors: The EPIC-InterAct study. Diabetologia (2013) 56:60–9. 10.1007/s00125-012-2715-x PMC4038917 23052052

[69] Zhu J Zong G Lu L Gan W Ji L Hu R. Association of genetic predisposition to obesity with type 2 diabetes risk in Han Chinese individuals. Diabetologia (2014) 57:1830–3. 10.1007/s00125-014-3308-7 24962670

[70] Werissa NA Piko P Fiatal S Kosa Z Sandor J Adany R. SNP-based genetic risk score modeling suggests no increased genetic susceptibility of the Roma population to type 2 diabetes mellitus. Genes (2019) 10:1–16. 10.3390/genes10110942 PMC6896051 31752367

[71] Zon SKR Reijneveld SA van der Most PJ Swertz MA Bültmann U Snieder H. The interaction of genetic predisposition and socioeconomic position with type 2 diabetes mellitus: Cross-sectional and longitudinal analyses from the lifelines cohort and biobank study. Psychosom Med (2018) 80:252–62. 10.1097/PSY.0000000000000562 29381659

[72] Kim DS Kim BC Daily JW Park S. High genetic risk scores for impaired insulin secretory capacity doubles the risk for type 2 diabetes in Asians and is exacerbated by Western-type diets. Diabetes Metab Res Rev (2018) 34:1–9. 10.1002/dmrr.2944 29048714

[73] Tuomilehto J Lindström J Eriksson JG Valle TT Hämäläinen H Ilanne-Parikka P. Prevention of type 2 diabetes mellitus by changes in lifestyle among subjects with impaired glucose tolerance. N Engl J Med (2001) 344:1343–50. 10.1056/NEJM200105033441801 11333990

[74] Gillies CL Abrams KR Lambert PC Cooper NJ Sutton AJ Hsu RT. Pharmacological and lifestyle interventions to prevent or delay type 2 diabetes in people with impaired glucose tolerance: Systematic review and meta-analysis. BMJ (Clin Res Ed) (2007) 334:299. 10.1136/bmj.39063.689375.55 PMC1796695 17237299

[75] Florez JC Jablonski KA Bayley N Pollin TI de Bakker PI Shuldiner AR. TCF7L2 polymorphisms and progression to diabetes in the Diabetes Prevention Program. N Engl J Med (2006) 355:241–50. 10.1056/NEJMoa062418 PMC1762036 16855264

[76] Hivert MF Jablonski KA Perreault L Saxena R McAteer JB Franks PW. Updated genetic score based on 34 confirmed type 2 diabetes Loci is associated with diabetes incidence and regression to normoglycemia in the diabetes prevention program. Diabetes (2011) 60:1340–8. 10.2337/db10-1119 PMC3064108 21378175

[77] Juonala M Magnussen CG Berenson GS Venn A Burns TL Sabin MA. Childhood adiposity, adult adiposity, and cardiovascular risk factors. N Engl J Med (2011) 365:1876–85. 10.1056/NEJMoa1010112 22087679

[78] Paulweber B Valensi P Lindström J Lalic NM Greaves CJ McKee M. A European evidence-based guideline for the prevention of type 2 diabetes. Horm Metab Res (2010) 42(Suppl 1):S3–36. 10.1055/s-0029-1240928 20391306

[79] Schnurr TM Jakupović H Carrasquilla GD Ängquist L Grarup N Sørensen TIA. Obesity, unfavourable lifestyle and genetic risk of type 2 diabetes: A case-cohort study. Diabetologia (2020) 63:1324–32. 10.1007/s00125-020-05140-5 32291466

[80] Li H Khor CC Fan J Lv J Yu C Guo Y. Genetic risk, adherence to a healthy lifestyle, and type 2 diabetes risk among 550,000 Chinese adults: Results from 2 independent Asian cohorts. Am J Clin Nutr (2020) 111:698–707. 10.1093/ajcn/nqz310 31974579 PMC7049535

[81] Ding M Ahmad S Qi L Hu Y Bhupathiraju SN Guasch-Ferré M. Additive and multiplicative interactions between genetic risk score and family history and lifestyle in relation to risk of Type 2 diabetes. Am J Epidemiol (2020) 189:445–60. 10.1093/aje/kwz251 PMC7462250 31647510

[82] Ericson U Hindy G Drake I Schulz CA Brunkwall L Hellstrand S. Dietary and genetic risk scores and incidence of type 2 diabetes. Genes Nutr (2018) 13:13. 10.1186/s12263-018-0599-1 29796113 PMC5956794

[83] Li SX Imamura F Schulze MB Zheng J Ye Z Agudo A. Interplay between genetic predisposition, macronutrient intake and type 2 diabetes incidence: Analysis within EPIC-InterAct across eight European countries. Diabetologia (2018) 61:1325–32. 10.1007/s00125-018-4586-2 PMC6445347 29549418

